# Overcoming multiple drug resistance mechanisms in medulloblastoma

**DOI:** 10.1186/2051-5960-2-57

**Published:** 2014-05-30

**Authors:** Ramadhan T Othman, Ioanna Kimishi, Tracey D Bradshaw, Lisa CD Storer, Andrey Korshunov, Stefan M Pfister, Richard G Grundy, Ian D Kerr, Beth Coyle

**Affiliations:** Children’s Brain Tumour Research Centre, Division of Child Health, Obstetrics and Gynaecology, School of Medicine, University of Nottingham, QMC, Nottingham, NG7 2UH UK; Centre for Biomolecular Sciences, School of Pharmacy, University of Nottingham, Nottingham, NG7 2UH UK; Clinical Cooperation Unit Neuropathology, German Cancer Research Center (DKFZ), Im Neuenheimer Feld 280, Heidelberg, Germany; Division of Pediatric Neurooncology, German Cancer Research Center (DKFZ), Im Neuenheimer Feld 580, 69120 Heidelberg, Germany; School of Life Sciences, University of Nottingham, QMC, Nottingham, NG7 2UH UK

**Keywords:** Medulloblastoma, ABCB1, MGMT, Etoposide, Temozolomide, Obatoclax

## Abstract

**Introduction:**

Medulloblastoma (MB) is the most common malignant paediatric brain tumour. Recurrence and progression of disease occurs in 15-20% of standard risk and 30-40% of high risk patients. We analysed whether circumvention of chemoresistance pathways (drug export, DNA repair and apoptotic inhibition) can restore chemotherapeutic efficacy in a panel of MB cell lines.

**Results:**

We demonstrate, by immunohistochemistry in patient tissue microarrays, that ABCB1 is expressed in 43% of tumours and is significantly associated with high-risk. We show that ABCB1, *O6*-methylguanine-DNA-methyltransferase (MGMT) and BCL2 family members are differentially expressed (by quantitative reverse transcription polymerase chain reaction, Western blotting and flow cytometry) in MB cell lines. Based on these findings, each pathway was then inhibited or circumvented and cell survival assessed using clonogenic assays. Inhibition of ABCB1 using vardenafil or verapamil resulted in a significant increase in sensitivity to etoposide in ABCB1-expressing MB cell lines. Sensitivity to temozolomide (TMZ) was MGMT-dependent, but two novel imidazotetrazine derivatives (N-3 sulfoxide and N-3 propargyl TMZ analogues) demonstrated ≥7 fold and ≥3 fold more potent cytotoxicity respectively compared to TMZ in MGMT-expressing MB cell lines. Activity of the BAD mimetic ABT-737 was BCL2A1 and ABCB1 dependent, whereas the pan-BCL2 inhibitor obatoclax was effective as a single cytotoxic agent irrespective of MCL1, BCL2, BCL2A1, or ABCB1 expression.

**Conclusions:**

ABCB1 is associated with high-risk MB; hence, inhibition of ABCB1 by vardenafil may represent a valid approach in these patients. Imidazotetrazine analogues of TMZ and the BH3 mimetic obatoclax are promising clinical candidates in drug resistant MB tumours expressing MGMT and BCL2 anti-apoptotic members respectively.

**Electronic supplementary material:**

The online version of this article (doi:10.1186/2051-5960-2-57) contains supplementary material, which is available to authorized users.

## Introduction

Medulloblastoma (MB) is the most frequent primary malignant brain tumour in children [1]. Currently the MB clinical staging system differentiates between standard-risk or high-risk disease depending on clinical findings and histologic subtype [2, 3]. Standard risk describes children older than 3 years of age at diagnosis with totally or near totally resected, non-disseminated disease and classic or desmoplastic histology. High-risk patients have either disseminated disease, incomplete resection or are of the large cell or anaplastic histologic subtypes [4, 5]. Treatment generally consists of gross total surgical excision combined with radiotherapy and chemotherapy; however 30-40% of high risk and 15-20% of standard risk patients develop recurrence of disease resulting in poor survival outcome [1, 3, 6–12]. Long term survivors suffer from treatment-associated endocrine, neurological, growth and neurocognitive complications that affect quality of life, in addition to having a substantial risk of secondary malignancy [13, 14]. From a chemotherapy perspective, primary MBs are mainly treated with combination chemotherapies that include: etoposide, methotrexate, cisplatin, lomustine, cyclophosphamide and vincristine [11, 15–17]. Recurrent and progressive tumours in children are treated with more intensive regimes frequently involving temozolomide (TMZ) and irinotecan although the prognosis for these patients is poor [1, 18–22].

Chemotherapy is now widely accepted as part of standard therapy for MB. The high frequency of recurrence, however, supports the hypothesis that a substantial number of patients have intrinsically drug resistant tumours [23]. Chemotherapy treatment failure is particularly relevant in children under three years of age who are preferentially treated with chemotherapy alone to minimise the adverse effect of radiotherapy on the developing brain [16, 24–26].

Classical multidrug resistance is often partly attributed to elevated expression of polyspecific ATP-dependent drug efflux pumps belonging to the ATP-binding cassette (ABC) transporter super family. The three best characterised are ABCB1 (also known as P-glycoprotein or MDR1), ABCG2 (Breast Cancer Resistance Protein) and ABCC1(multidrug resistance associated-protein 1 or MRP1) reviewed by Szakács *et al.*[27]. High expression of ABCB1 has previously been associated with chemoresistance and poor outcome in brain tumours including MB [28], glioma [29] and ependymoma (manuscript in preparation). We have also previously demonstrated that ABCB1 is expressed in a small subset of brain tumour cells and hypothesised that these cells are selected by current treatments resulting in relapse [30]. Since four of the drugs currently being used to treat primary and recurrent MB are ABCB1 substrates (etoposide, vincristine, methotrexate and irinotecan) [27] we would anticipate that overcoming/inhibiting ABCB1 could enhance the efficacy of current chemotherapy regimens.

Lomustine and TMZ are oral DNA alkylating cytotoxic drugs used for the treatment of primary and recurrent MB, respectively. The activity of both drugs depends on the absence of direct repair by *O6*-methylguanine-DNA-methyltransferase (MGMT) and proficient mismatch repair (MMR) [31]. Thus, over-expression of MGMT, a feature of more than half of MB patients in two recent studies [32, 33] can diminish the therapeutic efficacy of TMZ and lomustine. However, methylation of *O6*-guanine, the major cytotoxic lesion, comprises only 6% TMZ-DNA adducts [34], TMZ also methylates *N7*-guanine and *N3*- adenine, generating lesions which are repaired by base excision repair (BER) facilitated by the enzyme poly ADP ribose polymerase (PARP). Inhibition of PARP has been shown to enhance TMZ activity *in vitro* and *in vivo*[35, 36]. Recently, an alternative single agent approach has been shown to circumvent MGMT-mediated resistance in glioblastoma cell lines using novel imidazotetrazine TMZ analogues [37]. We wished to examine whether these agents were also able to effectively kill MB cells expressing high levels of MGMT.

Chemotherapy-induced cell cytotoxicity mainly occurs via the mitochondrial apoptotic pathway, regulated by the B-Cell lymphoma/leukaemia 2 (BCL2) family. Over-expression of anti-apoptotic BCL2 family proteins, such as BCL2, BCL-X_L_, MCL1 and BCL2A1, has been observed in many types of cancer including MB [38–41]. Hence, since the cloning of BCL2 25 years ago [42] many attempts have been made to target these pro-survival oncogenes therapeutically [43]. The most successful approach to target this pathway has been through the use of BCL2 homology 3 (BH3) mimetics; small molecules designed to mimic the BH3 domain found in pro-apoptotic members of the BCL-2 family. Recently two such small molecule inhibitors (ABT-737/ABT-263 and obatoclax) have entered clinical trial [44, 45]. We have previously tested ABT-737 on MB cell lines *in vitro* and although it was able to potentiate the action of cisplatin and etoposide, it proved ineffective as a single agent [46]. ABT-737 is described as a BAD mimetic since it shows the same binding to BCL2, BCL-X_L_ and BCL-w as this BH3 pro-apoptotic protein. We needed to determine whether the lack of ABT-737 efficacy as a single agent was due to the expression of additional BCL2 family members, targetable by the pan-inhibitor obatoclax, or the fact that ABT-737 has recently been demonstrated to be an ABCB1 substrate in chronic lymphocytic leukaemia [47].

In this study we set out to investigate three potential reasons for chemotherapy failure in MB using a combination of patient tissue microarrays and early passage cell lines. We show that whilst all three mechanisms do indeed contribute to MB chemoresistance, each of them can be effectively circumvented using a combination of novel agents (vardenafil, N3-propargyl and obatoclax).

## Materials and methods

### Patient characteristics

Clinical and histological data for the Nottingham retrospective cohort are outlined in the Additional file 1: Table S1. Clinical details of patients included in tissue microarrays (TMA) obtained from German Cancer Research Centre DKFZ were previously published by Dubuc *et al*. [48].

### Cell culture

Five new primary and recurrent (R) MB cell lines were derived as previously described [30] at the Children’s Brain Tumour Research Centre (CBTRC) (MED3, MED4, MED4R, MED5R and MED6) with approval from Local Research Ethics Committee. The tumours of origin were classified into molecular subtypes according to recent classification of MB subtypes [49–51]. Primary MB cell lines were cultured as monolayers and neurospheres as described previously [30]. The DAOY MB cell line was purchased from ATCC and cultured as recommended. UW228-3 was supplied by John R. Silber and grown as recommended [52]. A control Human Embryonic Kidney cell line (HEK-B1) stably expressing ABCB1 was provided as a gift by Rob Robey and cultured in DMEM with 10% serum and 500 μg/ml of G418.

### Immunohistochemistry

Immunohistochemistry (IHC) was performed using the Dako Envision Detection kit (DAKO REAL EnVision) [53]. Sections were counter-stained with haematoxylin (Leica Microsystems). As negative controls, adjacent or similar sections were processed with antibody diluent (Dako). The antibodies (Abs) used to stain the original patient samples with appropriate control for each Ab are summarised in Additional file 2: Table S2.

A total of 27 Nottingham MB patients diagnosed between January 1986 and January 2006 were analysed by IHC. Consent for tumour tissue use was taken in accordance with national tumour banking procedures (UK: 05/MRE/04/70). A second TMA comprising 233 patient samples was obtained from the DKFZ [48]. Both TMAs were stained with the anti-ABCB1 Ab (C219, 1:40; Calbiochem) and patients with clear evidence of cell membrane staining were scored as positive by three independent reviewers who were blinded to clinical and patient molecular variables.

### Detection of ABCB1 expression and inhibition of ABCB1 function by flow cytometry

To detect the percentage of ABCB1 expressing cells, 300,000-500,000 cells were incubated with 4E3 (anti-ABCB1; 1:20; Abcam) for 30 minutes at 37°C in fluorescent activated cell sorting (FACS) buffer (high glucose DMEM plus 5% BSA). The cells were then washed twice with FACS buffer and incubated with Alexa Fluor® 647 goat anti-mouse IgG (1: 1000; Invitrogen) for 30 minutes on ice in the dark. As a control, cells were stained with secondary Ab alone (Alexa Fluor 647; Invitrogen). Any unbound Ab was removed by washing the labelled cells twice with FACS buffer. To investigate ABCB1 function, cells were incubated with Rhodamine 123 (Rh123, Sigma; ABCB1 flourescent substrate), with and without ABCB1 inhibitors verapamil (VPL, Sigma), and vardenafil (TRC-Canada) at concentrations given in figure legends. Fluorescence was detected on a Cytomics FC500 flow cytometer (Beckman Coulter) and analysed using WinMDI version 2.8. Data presented are mean ± SEM of three independent experiments.

### Quantitative polymerase chain reaction

RNA extraction from cultured cells was performed using *mir*Vana miRNA Isolation kit (Ambion) and transcribed into cDNA using reverse transcriptase (Superscript III; Invitrogen). The resulting cDNA template was amplified and sequenced using primers previously published by Kumar *et al.*[54]. PCR products were sequenced on the forward and reverse strands at the DNA sequencing lab at the University of Nottingham.

Quantitative reverse transcription PCR (QRT-PCR) analysis was carried out using the CFX96 real time PCR machine (BIO-RAD) and iQ SYBR Green SuperMix (BIO-RAD) to assess the expression. Primers for MB molecular subtyping (WIF1, SFRP1, NPR3 and KCNA1) were designed and published by Zhao *et al.*[55]. ABCB1 primers were as published by Valera *et al.*[56]. The house-keeping gene GAPDH (Forward primer 5′ ATGTTCGTCATGGGTGTGAA 3′; reverse primer 5′GTCTTCTGGGTGGCAGTGAT 3′) was used as a control to normalise the data, as the cycle threshold value for GAPDH expression was consistent across the sample set. Relative values of transcripts were calculated using the Pfaffl equation [57]. Results are presented as mean ± SEM of three independent experiments, each experiment was performed in triplicate.

To identify *MYCC* and *MYCN* copy number elevation in MB samples, genomic DNA extraction from frozen cell pellets was performed as previously described [30]. Quantitative PCR (QPCR) analysis was carried out using the CFX96 real time PCR machine (BIO-RAD) and iQ SYBR Green SuperMix (BIO-RAD). DNA isolated from D458 cells was used as a positive control for *MYCC* and a previously described sample of anaplastic astrocytoma with *MYCN* amplification was used as a positive control for MYCN [58]. *MYCC* and *MYCN* copy numbers were measured relative to the endogenous control *RPLP0* and results for each sample were normalised to the copy number of diploid human DNA in Pfaffl equation [57]. Primer sequences have been previously published by Ryan et al. [59].

### Western blotting

SDS PAGE and Western blotting were performed as previously described [46]. Blots were probed with the following Abs: mouse anti-ABCB1 (anti-C219 mouse monoclonal Ab; Calbiochem1:100), anti-MGMT (Millipore 1:100), rabbit anti-BCL2 (Cell Signaling clone 50E3 1:1000), rabbit anti BCL2A1 (Cell Signaling 1:1000), Rabbit anti-MCL1 (Santa Cruz 1:500) and either mouse anti-GAPDH (Sigma) or rabbit anti-β-tubulin (Cell Signalling Technology) 1:1000 as loading controls. All primary Abs were detected using goat anti-mouse/rabbit IgG HRP-linked secondary Ab (Cell Signalling Technology 1:2000) and enhanced chemoluminescence (GE Health Care Life Science) performed according to the manufacturer’s protocol.

### Clonogenic assay

Cell growth inhibition was estimated after treatment of single cells in 6 well plates. Cells (150–200) were seeded into 6 well plates, and allowed to adhere (4–8 hours) before drug treatment. At the outset the clonogenic range was established for each drug and used to derive IC_50_ drug concentrations. The range of drug concentrations tested and the length of the treatment period was dependent on the drug being tested. Cells were incubated with: 0–8 μM etoposide (Sigma) for 2 hours; 0–50 μM ABT-737 (Benzamide; Selleckchem) for 24 hours; 0–3 μM obatoclax mesylate (GX15-070; Geminx) for 24 hours; 0–800 μM (MGMT+) or 0–100 μM (MGMT-) TMZ for 2 hours; and up to 200 μM N-3 sulfoxide and N-3 propargyl for 2 hours. Inhibitors were added for the same length of time as the drug, either in the same well or separately to assess toxicity. For experiments to investigate the role of ABCB1 in mediating drug resistance; verapamil (10, 20 μM Sigma) and vardenafil (5, 10 μM Toronto Research Chemicals) were tested. To investigate potentiation of TMZ or N3-propargyl by circumvention of BER the PARP inhibitor rucaparib (0.4 μM AG 014699; Selleckchem) was tested. Following drug +/− inhibitor addition, the cultures were then maintained at 37°C in a humidified atmosphere containing 5% v/v CO_2_, and allowed to grow for 6 (MED1), 7 (DAOY and UW228-3), or 12 (MED3) days depending on the cell line’s doubling time. Colonies were fixed with 4% w/v paraformaldehyde and stained with 0.1% w/v crystal violet (Sigma). Colonies that contained more than 50 cells (≥6 doublings) were counted. The colonies were double-scored (one of the scorers being blind to the culture conditions). Clonogenic assays were repeated at least three times as independent experiments, each with internal duplicate cultures. The percentage of clonogenic survival was expressed relative to vehicle controls.

### Statistical analysis

Log-rank analysis on Kaplan–Meier curves determined the significance of overall survival (OS) and progression free survival (PFS) using SPSS version 21 statistical software (IBM). Differences between pairs of groups were determined by the Student’s *t*-test or Chi-squared test using GraphPad prism version 5.00 (San Diego California, USA) or IBM SPSS version 21 respectively. Response to TMZ and TMZ analogues between groups was assessed using one way ANOVA. *P* values < 0.05 were considered significant.

## Results

### ABCB1 expression is associated with high risk MB

**Figure 1:**
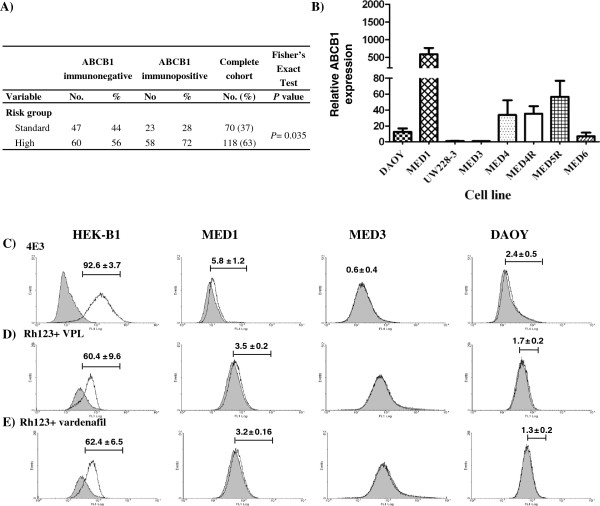
**ABCB1 expression and functional analysis. (A)** Membranous ABCB1 expression was scored across the DKFZ TMA and correlated with treatment risk group in 188 patients >3-17 years old. ABCB1 was significantly more likely (P ≤ 0.035) to be expressed in samples from high risk patients. **(B)** Relative expression of ABCB1 was calculated for each cell line using MED3 as a calibrator and GAPDH as a house keeping gene in the Pfaffl equation [57]. ABCB1 is expressed in 6/8 MB cell lines. Data are expressed as the mean ± SEM of three experiments, each performed in triplicate. **(C)** Flow cytometry analysis of ABCB1 cell surface expression was carried out on MED1, MED3, DAOY and HEK-B1 control cell lines stained with anti-ABCB1 antibody (clone 4E3; unfilled histogram) or secondary antibody alone (filled histogram). Although only 5.8% of MED1 cells are positive for ABCB1 some cells expressed very high levels whereas MED3 showed no expression of ABCB1 (0.6 ± 0.4%). Representative dotplots can be found in Additional file 11: Figure S7. **(D, E)** ABCB1 function was tested by flow cytometry. Cells were incubated with either Rh123 alone (filled histogram) or in combination with VPL **(D)** or vardenafil **(E)** shown as unfilled histograms in each case. Inhibition of ABCB1 function was observed in 3.5% of MED1 cells consistent with the low% of cells expressing ABCB1. Similarly, there was no inhibition of function in MED3 cell line. Results in **(C-E)** represent mean ± SEM of three independent experiments.

There is evidence supporting a role for MGMT and the BCL2 family in MB chemotherapeutic response; however, the evidence supporting ABCB1 was more equivocal [28, 33, 41]. We therefore set out to identify whether there were clinical correlates of ABCB1 expression in a retrospective Nottingham MB patient TMA. In total 12 of 27 samples (44%) were positive for ABCB1 expression, and the majority of these were high risk patients, but this relationship was not statistically significant (*P* ≥ 0.05), presumably due to the small number of patient samples. We then analysed ABCB1 expression across a larger German DKFZ TMA. On this array 42% (99/233) of samples were positive for ABCB1 expression and ABCB1 was significantly more likely (*P =* 0.035) to be expressed in samples from high risk patients (Figure 1A). There was no significant difference between age, gender, resection status, and recurrence group of the two studies (*P* > 0.05, Additional file 3: Table S3). In each case positive samples contained a subpopulation of cells with membranous ABCB1 expression (0.1-11%). This high frequency of ABCB1 expression in MB patients and association with high risk clearly justified its inclusion in our chemoresistance analyses of patient derived cell lines.

### Characterisation of MB cell lines and their tumour of origin

To investigate the mechanisms of drug resistance in MB, we have adopted an approach that involves establishing cell lines from patients’ primary and recurrent tumours [30]. In the current work, we describe 6 newly derived MB cell lines (including the previously described MED1) [30]. The clinical characteristics of these patients are presented in supplementary Additional file 4: Table S4. The tumours from which the cell lines were derived have been assigned to MB molecular subtypes [60, 61] using IHC for β-catenin, Gli1, NPR3 and KCNA1 (Additional file 5: Figure S1), and sequencing of the β-catenin gene (*CTNNB1*). The corresponding cell lines were sub-grouped by comparing expression of representative genes [WIF1 (WNT), SFRP (SHH), NPR3 (Group 3), and KCNA1 (Group 4)] to foetal cerebellum (Additional file 6: Figure S2, [55]). *MYCC* and *MYCN* copy number were determined (Additional file 7: Figure S3) and β-catenin sub-grouping was again confirmed by sequencing *CTNNB1* (Additional file 8: Figure S4). These data are summarised in Table 1. Our data therefore suggest that MED1 is derived from a large cell anaplastic Group 4 tumour with MYCN amplification. MED3 is derived from a classical Group 3 tumour, MED4, and MED4R result from Group 3 tumours with MYCN gain. In each case the cell lines maintain the characteristics of their tumour of origin. MED5R and MED6 are derived from WNT subtypes (β-catenin positive) large cell anaplastic and classical tumours respectively. MED5R and MED6 have heterozygous mutations (TCT-TTT) at codons 33 and 37 (of *CTNNB1*) respectively. The MED5R cell line maintained the mutation in culture whereas the MED6 cell line reverted to wild type (Additional file 8: Figure S4). We also established that UW228-3 cells are NPR3 positive with MYCC gain. DAOY cells, on the other hand proved difficult to classify, although, have been classified recently as SHH by Pambid *et al.*[62].

**Table 1:** **Characterisation of MB original patient samples and their derived cell lines**

Name	Tumour						Cell lines			
	Histology	IHC MB subtype marker	***CTNNB1*** mutated	IHC ABCB1 %	IHC MGMT	MB subtype	Subtype QRTPCR	MYC status	***CTNNB1*** mutated	MB subtype
MED 1	LC/A	KCNA1	No	11.2	-	Group 4	**KCNA1 positive**	**MYCN Amplified**	**No**	**Group 4**
MED 3	Classical	Moderate NPR3	No	0	+	Group 3	**NPR3 positive**	**Normal**	**No**	**Group 3**
MED4	LC/A	NPR3	No	1.9	+	Group 3	**NPR3 positive**	**MYCN gain**	**No**	**Group 3**
MED4R	LC/A	NPR3	No	4.5	+	Group 3	**NPR3 positive**	**MYCN gain**	**No**	**Group 3**
MED5R	LC/A	β-catenin	Yes	6.2	+	WNT*	**NPR3 positive**	**Normal**	**Yes**	**WNT**
MED6	Classical	β-catenin	Yes	1.2	+	WNT*	**NPR3 positive**	**Normal**	**No**	**Group 3**
UW228-3	Classical	NA	NA	NA	NA	Group 3	**NPR3 positive**	**MYCC gain**	**No**	**Group 3**
DAOY	Desmoplastic	NA	NA	NA	NA	SHH?	**None**	**Normal**	**No**	**SHH**[62]**

*Abbreviation:* LC/A = large cell/anaplastic medulloblastoma, R=recurrent, IHC = immunohistochemistry, WNT = β-catenin positive, Group 3 = NPR3 positive, Group 4= KCNA1 positive, MED4 and MED4R are from the same patient.

*Frozen tissue from MED5R and MED6 has CTNNB1 mutation at codon 33 and 37 respectively. NA= tumour material not available. **DAOY cell line was characterised as SHH by Pambid *et al*. [62].

### ABCB1 expression and function is maintained in MB cell lines

ABCB1 expression was assessed in each of the tumours of origin by IHC (Additional file 9: Figure S5). The percentage of ABCB1 expressing cells ranged from ~1% in the MED6 to 11.2% in the MED1 tumour of origin (Table 1). ABCB1 expression was then assessed in the 6 CBTRC cell lines derived from these MBs as well as two more generally studied MB cell lines, DAOY and UW228-3, by QRT-PCR (Figure 1B). In common with the tumour of origin, MED1 cells expressed the highest levels of ABCB1 mRNA (Figure 1B) and glycosylated ABCB1 protein (Additional file 10: Figure S6), thus confirming maintenance of ABCB1 expression in culture. MED4, MED4R and MED5R demonstrated intermediate ABCB1 expression whereas MED3 cells expressed negligible mRNA and protein, again correlating with protein expression levels in primary tumours (Figure 1B, Additional file 10: Figure S6, Table 1). DAOY cells showed intermediate ABCB1 expression, whereas UW228-3 cells expressed low ABCB1 mRNA and protein levels. Three cell lines demonstrating high (MED1), intermediate (DAOY) and low (MED3) ABCB1 expression were further investigated for cell surface expression and function of ABCB1 by flow cytometry and compared to HEK-B1 cells stably expressing ABCB1 (Figure 1C-E). For MED1 5.8 ± 1.2% cells were positive for ABCB1 with some cells showing very high expression (Figure 1C, Additional file 11: Figure S7). MED3 cells demonstrated negligible ABCB1 expression (0.6 ± 0.4%). Within the DAOY cell population 2.4 ± 0.5% cells expressed consistently low ABCB1 levels (See Figure 1C, Additional file 11: Figure S7). ABCB1 function was investigated with the Rh123 extrusion assay. Extrusion of Rh123 in MED1 was inhibited by 20 μM VPL (right shifted cell histogram) and 10 μM vardenafil (Figure 1D,E) in ~3.5% of cells. Thus both agents are able to inhibit Rh123 extrusion in 60% of ABCB1 expressing (3.5/5.8) cells. Similar levels of inhibition (54-66%) were observed with both inhibitors in HEK-B1 and DAOY cells. MED3 cells failed to extrude Rh123 consistent with lack of ABCB1 cell surface expression.

### Co-treatment with ABCB1 inhibitors VPL and vardenafil results in increased etoposide cytotoxicity

**Figure 2:**
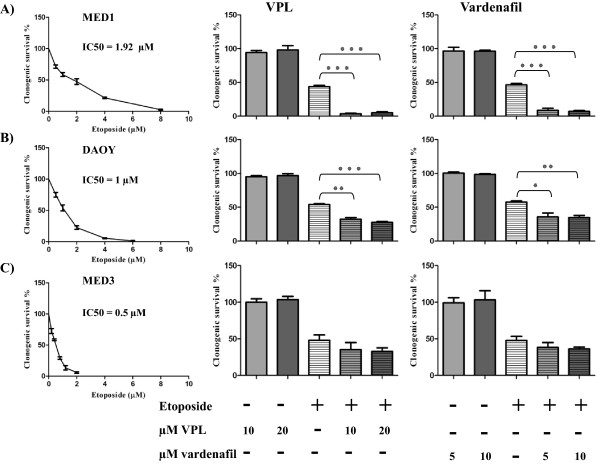
**Clonogenic survival analysis of MB cells treated with etoposide in the presence of ABCB1 inhibitors.** The left panel shows the effect of increasing etoposide concentrations on clonogenic survival (100% representing solvent control). The middle and right panels represent clonogenic survival in the presence of etoposide (IC_50_ concentrations derived from dose–response data) in combination with VPL (10 and 20 μM) or vardenafil (5 and 10 μM) respectively. **(A)** MED1 clonogenic survival is significantly inhibited in cells co-treated with etoposide and VPL or vardenafil (unpaired student’s *t*-test, ٭٭٭*P* < 0.0001 in all cases). **(B)** There was also a significant increase in etoposide cytotoxicity in DAOY cells when combined with VPL and vardenafil (unpaired student’s *t*-test, ٭٭*P* < 0.001; ٭*P* = 0.02). **(C)** No significant inhibition was observed in the MED3 cell line. Data represent mean ± SEM of n ≥ 3 experiments, each experiment was performed in duplicate.

To further investigate ABCB1 function *in vitro,* clonogenic survival was assessed in ABCB1 negative MED3 and 2 ABCB1 positive (MED1 and DAOY) MB cell lines in response to etoposide alone or in combination with the ABCB1 inhibitors VPL and vardenafil. The IC_50_ concentrations for etoposide reflected the expression levels of ABCB1, with MED1 demonstrating the greatest degree of resistance to etoposide (MED1 1.92 μM, DAOY 1 μM, and MED3 0.5 μM) (Figure 2). Both ABCB1-expressing cell lines demonstrated significantly increased etoposide sensitivity when cells were treated with etoposide at the IC_50_ concentration plus inhibitors VPL or vardenafil compared to cells treated with etoposide alone (MED1 *P* < 0.0001 in all cases, Figure 2A; DAOY *P* < 0.001 in all cases except 5 μM vardenafil *P* = 0.02; Figure 2B). Conversely, no significant potentiation of cell cytotoxicity was observed when MED3 cells were treated with etoposide plus ABCB1 inhibitors (*P* > 0.05; Figure 2C). These results confirm that etoposide cytotoxicity in MB cell lines is significantly influenced by ABCB1 expression. Moreover, our *in vitro* assays confirm that vardenafil significantly inhibits ABCB1.

### Obatoclax-induced apoptosis in MB cell lines is independent of MCL1, BCL2A1 and ABCB1 expression

**Figure 3:**
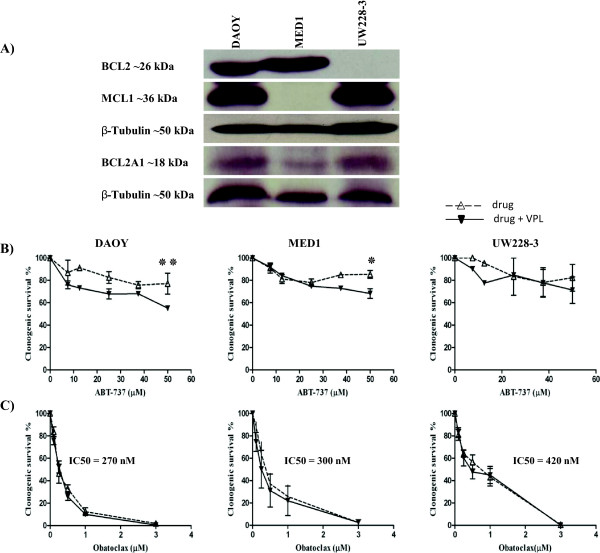
**BCL2 family protein expression and functional analysis.** BCL2A1 and ABCB1 limit response to ABT-737 but not obatoclax. **(A)** DAOY, UW228-3 and MED1 cells were analysed by Western blotting for expression of anti-apoptotic BCL2 protein family member, BCL2 is expressed in DAOY and MED-1 whereas no expression was observed in UW228-3 cells. MCL1 is expressed in DAOY and UW228-3 whereas MED-1 displayed no expression. BCL2A1 was expressed at a low level in all three cell lines. β-tubulin served as a loading control. **(B, C)** Clonogenic survival of MB cells in the presence of ABT-737 **(B)** or obatoclax **(C)** alone (dotted line) or in combination with 10 μM VPL (solid lines). Although IC_50_ values were not reached, significant potentiation of ABT 737 function was observed at 50 μM concentration in combination with VPL in DAOY and MED1 cell lines (unpaired student’s *t*-test, ٭٭*P* = 0.03; ٭*P* = 0.02 respectively). Conversely, obatoclax IC_50_ values were in the nanomolar range for all 3 cell lines and there was no potentiation when combined with VPL. Results from B and C represent mean ± SEM of n ≥ 3 experiments, all performed in duplicate.

We have previously shown that MB cell lines express MCL1 and BCL2 anti-apoptotic proteins, and that the BH3 mimetic ABT-737, although able to potentiate activity of chemotherapeutic drugs, failed to induce apoptosis as a single agent in MB cell lines [46]. The lack of efficacy of ABT-737 could not be explained by BCL2 and MCL1 expression alone. Therefore we sought to further investigate the efficacy of ABT-737 against MB cell lines and compare it to the new anti-BCL2 drug obatoclax. We first looked at anti-apoptotic (BCL2, MCL1 and BCL2A1) protein expression in three representative MB cell lines, including UW228-3 since this was used in our previous study (Figure 3A). Whereas BCL2 and MCL1 were differentially expressed, consistent with our previous report [46], all 3 cell lines expressed BCL2A1. Previous studies on leukaemia cell lines have shown that ABT-737 is a substrate for ABCB1 [47], therefore the pro-apoptotic activity of ABT-737 and obatoclax were tested on these 3 cell lines with and without the ABCB1 inhibitor VPL (Figure 3B, C). ABT-737 was tested up to maximum 50 μM concentration (DMSO > 0.1%) but it was only able to reduce cell colony formation by approximately 20%, 15% and 20% in DAOY, MED1 and UW228-3 respectively. There was a small but significant increase in ABT-737 efficacy when ABCB1 positive MB cells were treated with ABT-737 (50 μM) plus 10 μM of VPL (*P* < 0.03 for MED1 and *P* < 0.02 for DAOY; Figure 3B) but no effect of VPL was found on ABCB1 negative UW228-3 cells (*P* ≥ 0.05). Obatoclax caused significantly decreased clonogenic cell survival resulting in less than 2% clonogenic survival at the highest concentration (3 μM) in all 3 cell lines (Figure 3C). The IC_50_ of obatoclax in DAOY, MED1 and UW228-3 was 0.27, 0.3 and 0.42 μM respectively. Most importantly, there was no significant change in colony formation following treatment of cells with obatoclax and VPL (*P* > 0.05; Figure 3C). Our *in vitro* results suggest that obatoclax is not an ABCB1 substrate; can inhibit BCL2, MCL1 and BCL2A1, and therefore, is more effective than ABT-737 since it can function as a single agent.

### Growth inhibition by the two novel TMZ analogues (N-3 sulfoxide and N-3 propargyl) is MGMT independent

**Figure 4:**
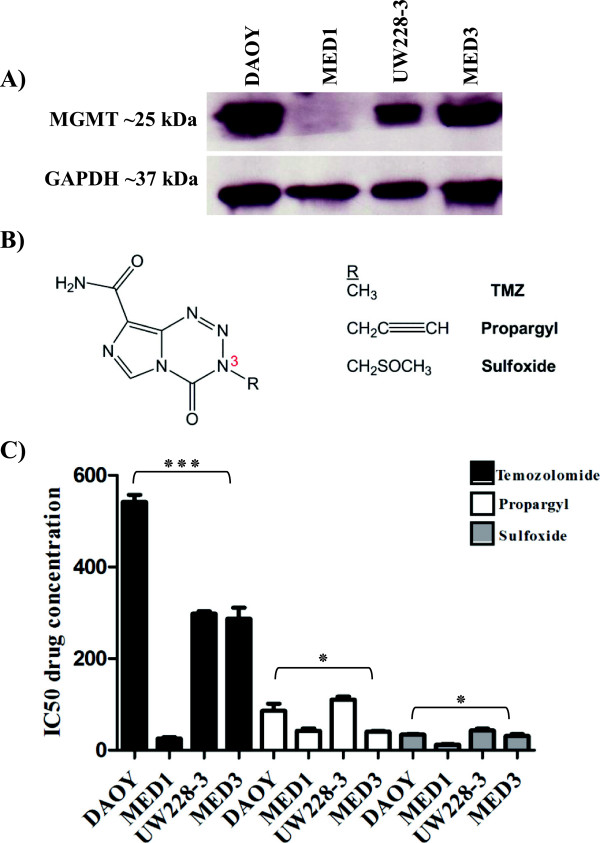
**MGMT expression and response to TMZ, propargyl and sulfoxide imidazotetrazine analogues. (A)** Relative levels of MGMT expression were determined by Western blotting. DAOY, UW228-3 and MED3 cells showed high expression of MGMT while no expression was observed in MED1 cells. **(B)** Chemical structure of TMZ, propargyl and sulfoxide, shows different N3 groups. **(C)** Clonogenic survival was determined as in Figures 2, and 3, the data represent the IC_50_ values (mean ± SEM, n ≥ 3) TMZ inhibited survival in an MGMT-status dependent manner (IC_50_ at 290–540 μM in MGMT positive cells (DAOY, UW228-3 and MED3) compared to 31 μM in MGMT negative MED1 cells). Propargyl and sulfoxide derivatives of TMZ inhibited survival at 40–80 and 20–40 μM respectively in all cell lines, showing no dependence on MGMT expression (unpaired student’s *t*-test, ٭٭٭*P* < 0.0001; ٭*P* < 0.05).

TMZ cytotoxicity depends on the absence of MGMT enzyme and functioning MMR [31]. MGMT expression was assessed in all tumours of origin for our cell lines by IHC (Additional file 12: Figure S8, Table 1). Only MED1 was MGMT negative, with all other samples showing strong MGMT expression. DAOY, UW228-3 and MED3 MB cell lines all show high MGMT expression while MED1 cells show no MGMT expression by Western blotting, consistent with the observed expression in primary tissue samples from the corresponding tumours (Figure 4A, Additional file 12: Figure S8). The efficacy of TMZ and two novel imidazotetrazine TMZ analogues (N-3 sulfoxide and N-3 propargyl; Figure 4B) were compared using the clonogenic assay. Clonogenic survival was concentration-dependent in all 4 cell lines. DAOY, UW228-3 and MED3 were resistant to TMZ (IC_50_ = 290–540 μM; Figure 4C); in contrast MED1 cells were sensitive (IC_50_ = 31 μM; Figure 4C). All MB cell lines were sensitive to sulfoxide and propargyl analogues independent of MGMT expression (sulfoxide IC_50_ 15–40 μM whereas propargyl IC_50_ 42.4-96.3 μM; Figure 4C). To investigate whether BER was also able to modulate response we determined the IC_50_ concentrations for TMZ and the propargyl analogue when cells were treated in combination with the PARP inhibitor rucaparib (Additional file 13: Figure S9). There was considerable chemosensitisation of TMZ by rucaparib in MGMT expressing DAOY, MED3 and UW228-3 cell lines (1.9 fold *P* = 0.02, 2.7 fold *P* = 0.002 and 2.2 fold *P* = 0.029 respectively; Table 2, Additional file 13: Figure S9). There was however no potentiation of propargyl cytotoxicity in combination with rucaparib (Table 2, Additional file 13: Figure S9). These results suggest that in MB cells cytotoxicity induced by the N-3 propargyl analogue is independent of MGMT and BER.

**Table 2:** **Toxicity and efficacy of TMZ and propargyl in combination with PARP inhibitor rucaparib in MB cell lines**

Cell line	IC_50_TMZ	IC_50_TMZ + Rucaparib	PF	IC_50_Propargyl	IC_50_Propargyl + Rucaparib	PF
DAOY	540 ± 70.2	253 ± 35.1	1.9*	68.3 ± 26.2	47.9 ± 4.6	1.4
MED1	31 ± 12.4	26.8 ± 10.9	1.2	42.2 ± 9.5	43.4 ± 14	1.0
UW228-3	290 ± 14.8	129.5 ± 12	2.2*	96.3 ± 21.2	94.3 ± 38	1.0
MED3	332.9 ± 81.8	124.7 ± 55.3	2.7**	53.6 ± 16.8	56.3 ± 26.6	1.0

*Abbreviation:* IC_50_ = concentration resulting in 50% inhibition. PF = potentiation factor. ٭٭*P* = 0.002; ٭*P* < 0.05 (unpaired student’s *t*-test). Result of n ≥ 3 experiments, all performed in duplicate.

## Discussion

Benefit of adjuvant chemotherapy has been demonstrated in children [10] particularly those under 3 years of age with desmoplastic MB [3, 9, 10, 15, 16, 25]. However, particularly in high risk disease and in children under 3 years of age with classic and anaplastic MB, chemotherapy failure due to drug resistance is a recognised limitation to cure. There are many aspects to chemotherapy resistance with protective mechanisms operating at each stage of the cytotoxic process. We have sought here to investigate three basic mechanisms that are key to effective chemotherapy cytotoxicity and resistance. Firstly, chemotherapeutic drugs must remain in the tumour cell long enough to damage DNA. Secondly, this damage must not be repaired. Thirdly, downstream apoptosis mechanisms must be active. By studying a set of MB cell lines we have demonstrated that all of these processes reducing cytotoxicity are active, but can be effectively inhibited or circumvented. Firstly, we have demonstrated that > 40% of MB tumours express the ABCB1 multi-drug transporter, which is able to export many of the currently used chemotherapeutic drugs. There was no difference in ABCB1 expression between the 4 molecular subtypes (data not shown). Importantly, our *in vitro* data shows that export by ABCB1 could be inhibited resulting in increased cytotoxicity. Secondly, we have shown that although MGMT is expressed in the majority of MB patients’ tumours, and is able to directly repair the effects of DNA alkylating agents it cannot repair the effects of two novel imidazotetrazine derivatives of TMZ (N-3 sulfoxide and N-3 propargyl) in MB cells. Thirdly, our data indicate that, despite the expression of several BCL2 anti-apoptotic family members, the BH3 mimetic obatoclax is effective as a single agent in MB.

Multidrug transporters serve as a major mechanism of defence in cancer chemotherapy. We hypothesise that the treatment of MB with current chemotherapeutic drugs, although effective as adjuvant therapy, leads to selection of an ABCB1 transporter expressing sub-populations of cells that in time can give rise to a more resistant tumour [63]. Importantly we have shown that in the DKFZ array ABCB1 expression is associated with patients that fall into the high risk category, which despite more aggressive therapy have a poorer outcome [2, 6, 7]. The concentration of etoposide used to achieve an IC_50_ in the ABCB1 positive MED1 cell line was slightly above the concentration used in clinic [64] while the MED3 IC_50_ was below the dose limiting toxicity. The data suggest however that the application of drugs that circumvent ABC transporters could therefore represent a valid addition to current MB chemotherapy regimens, particularly in high risk MB. We have used two inhibitors to overcome ABCB1 resistance in MB cells. VPL (a calcium channel blocker) is a nonspecific ABCB1 inhibitor and its low affinity to ABCB1 necessitated use of a high dose to block ABCB1 in clinical trials, resulting in unacceptable cardiac toxicity [65]. Vardenafil is a phosphodiesterase type 5 inhibitor used to treat children and infants with pulmonary arterial hypertension and portal hypertension with minimal adverse effects at concentrations above those indicated in our study [66, 67]. It has been previously shown that vardenafil can specifically block ABCB1 transporter function and enhance blood–brain and blood–brain tumour barrier permeability thereby enhancing delivery of herceptin to the brain in mice bearing intracranial breast and lung cancer [68, 69]. Our results showed that vardenafil enhances etoposide cytotoxicity in ABCB1-expressing MB cell lines. The activity of vardenafil in each of our assays was equivalent to that of the non-specific ABCB1 inhibitor verapamil. Thus, vardenafil is a good potential candidate for combination with current chemotherapeutics in patients with MB and should now be tested *in vivo* in pre-clinical models.

Many efforts have been made towards development of small molecules able to overcome the anti-apoptotic activity of anti-apoptotic BCL2 family proteins. ABT-737 can overcome BCL2 and BCL-X_L_ and is currently in phase 1 clinical trial in small cell lung cancer (SCLC) and chronic lymphocytic leukaemia [45, 70]. Although effective as a single agent in SCLC and leukaemia, ABT-737 was not able to induce apoptosis as a single agent in MB cells [46, 71, 72]. It has been previously shown that ABT-737 function can be limited by BCL2A1 and MCL1 protein expression [73–75]. Additionally, ABT-737 has been demonstrated to be a substrate for ABCB1 and ABCC1 [47, 76]. Our data suggest that ABT-737 induction of apoptosis in MB is dependent of both BCL2A1 and ABCB1 protein expression. Using an ABCB1 inhibitor did significantly potentiate the activity of ABT-737 in MB cells; however the IC_50_ concentration was still not reached and these concentrations would result in significant toxicity *in vivo*[77, 78]. Obatoclax, on the other hand readily induced apoptosis, regardless of BCL2A1 and ABCB1 protein expression, at concentrations below the dose limiting toxicity concentration observed in phase 1 clinical trial [39]. Obatoclax has been used in a phase 1 clinical trial on advanced leukemic patients where it showed minimum toxicity and one patient had complete remission for 8 months [44]. We believe obatoclax is a better candidate than ABT-737 and may therefore represent a viable therapeutic option in MB patients.

TMZ and Lomustine are alkylating agents commonly used in clinic, however, the DNA damage cause by these drugs can be repaired by MGMT [31]. Notably only the IC_50_ concentration in the MGMT negative cell lines (MED1) was below the maximum tolerated dose of TMZ used in clinical trial [79]. Two novel imidazotetrazine analogues N-3 sulfoxide and N-3 propargyl TMZ derivatives used in this project were developed by Pharminox in order to overcome direct DNA repair by MGMT [31]. It is hypothesised that propargyl and sulfoxide analogues deliver cytotoxic lesions to *O6*-guanine which cannot be removed by MGMT and both analogues have been shown to cause double DNA strand breaks leading to death in glioma cells. Overcoming MGMT resistance in MB may be reliant on MB tumours being proficient in mismatch repair. This is potentially not an issue since MMR proficiency was recently demonstrated in 74/74 primary MB tumours in a study by von Bueren *et al.*[33]. In addition to MGMT repair of *O6*-methylguanine, TMZ methylation at *N7*-methylguanine and *N3*- methyladenine purines can also be repaired by PARP through BER. Our data demonstrate that sulfoxide and propargyl analogues are cytotoxic to all 4 MB cell lines regardless of MGMT expression and that, unlike TMZ, the efficacy of the propargyl analogue cannot be potentiated by the PARP inhibitor rucaparib. This suggests that putative propargyl modification at *N3* and *N7* positions may not be recognized by PARP, or cannot be repaired by BER. Stability studies of the 2 analogues have shown that N3-propargyl is more stable than TMZ in plasma (100 minutes versus 30 minutes respectively); however, the N3-sulfoxide analogue is extremely unstable in plasma and hence unsuitable as a clinical candidate. Hence, propargyl could be a good substitute for TMZ in recurrent and progressive MBs and may also be effective in primary tumours.

## Conclusions

We have tested three mechanisms of resistance to chemotherapy mediated by ABCB1, MGMT and anti-apoptotic BCL2 family members in MB cell lines. Targeting these factors either by using an ABCB1inhibitor (vardenafil), a novel agent that can circumvent DNA repair (N3-propargyl analogue of TMZ) or a BH3 mimetic to inhibit BCL2 anti-apoptotic proteins (obatoclax) could all be viable therapeutic routes in this malignant tumour in children. Our study of a small subset of MB cell lines, however, indicates that these resistance mechanisms are not mutually exclusive. Hence, the next stage should not only be to test the efficacy of these agents in *in vivo* models but also to use them in combination. We believe that such an approach will help to redefine MB as a more chemo-sensitive disease.

## Electronic supplementary material

Additional file 1: Table S1: Clinicopathological characteristic of MB patients included in Nottingham TMA. (DOCX 14 KB)

Additional file 2: Table S2: Antibodies used in the IHC analysis. (DOCX 15 KB)

Additional file 3: Table S3: Correlation of ABCB1 expression with clinicopathological characteristics of DKFZ + Nottingham TMA cohorts. (DOCX 15 KB)

Additional file 4: Table S4: Clinical characteristics of the 5 patients from whom 6 new MB cell lines were derive. (DOC 39 KB)

Additional file 5: Figure S1: Patterns of MB marker subtype immunostaining on each patient’s original tissue sample. (JPEG 1 MB)

Additional file 6: Figure S2: Molecular sub-classification of the 8 MB cell lines. (PDF 160 KB)

Additional file 7: Figure S3: Elevated *MYCC* and *MYCN* copy numbers in MB cell lines. (PDF 323 KB)

Additional file 8: Figure S4: β-catenin (*CTNNB1*) sequencing in MED5R and MED6 tumours and cell lines. (PDF 32 KB)

Additional file 9: Figure S5: ABCB1 expression in original patient tumours. (JPEG 695 KB)

Additional file 10: Figure S6: MED1 cells show high ABCB1 protein expression. (JPEG 185 KB)

Additional file 11: Figure S7: ABCB1 expression is found in a small subpopulation of cells. (PDF 37 KB)

Additional file 12: Figure S8: MGMT expression in the original patient tumours. (JPEG 768 KB)

Additional file 13: Figure S9: Chemosensitization of TMZ and propargyl by rucaparib in MB cell lines. (PDF 133 KB)

## Abbreviations

MB: Medulloblastoma
MGMT: O6-methylguanine-DNA-methyltransferase
TMZ: Temozolomide
ABC: ATP-binding cassette
ABCB1: ATP-Binding Cassette, Sub-Family B (MDR/TAP), Member 1, P-glycoprotein or MDR1
MMR: Mismatch repair
PARP: Poly ADP ribose polymerase
BER: Base excision repair
BCL2: B-cell lymphoma/leukaemia 2
BH3: BCL2 homology 3
HEK: Human embryonic kidney cell line
IHC: Immunohistochemistry
FACS: Fluorescent activated cell sorting
Ab: Antibody
Rh123: Rhodamine 123
VPL: Verapamil
SEM: Standard error of the mean
QPCR: Quantitative polymerase chain reaction
OS: Overall survival
PFS: Progression free survival
SDS PAGE: Sodium dodecyl sulfate poly-acrylamide gel electrophoresis.
