# Supplementary material for: Overcoming multiple drug resistance mechanisms in medulloblastoma
Source: Acta Neuropathol Commun. 2014 May 30;2:57. doi: 10.1186/2051-5960-2-57 (PMC4229867; doi:10.1186/2051-5960-2-57)
Supplement: Supplementary file 1 — Additional file 1: Table S1: Clinicopathological characteristic of MB patients included in Nottingham TMA. (DOCX 14 KB) [file 40478_2014_133_MOESM1_ESM.docx]

**Additional file 1: Table S1** Clinicopathological characteristic of MB patients included in Nottingham TMA

| **Variable** | **No** | **%** |
| --- | --- | --- |
| **Gender** |  |  |
| F | 7 | 26 |
| M | 20 | 74 |
| **Age, years** |  |  |
| <3 | 4 | 15 |
| ≥3 | 23 | 85 |
| **Metastatic status** |  | |
| M- | 16 | 59 |
| M+ | 10 | 37 |
| Unknown | 1 | 4 |
| **Resection status** |  | |
| Complete | 11 | 41 |
| Incomplete | 14 | 52 |
| Unknown | 2 | 7 |
| **Histology** |  | |
| Classical | 12 | 44 |
| Desmoplastic | 9 | 35 |
| LC/A | 5 | 17 |
| unknown | 1 | 4 |
| **Recurrence** |  | |
| Yes | 20 | 74 |
| No | 7 | 26 |
| **Risk group*** |  |  |
| Standard | 5 | 19 |
| High | 20 | 74 |
| Unknown | 2 | 7 |
| **Status** |  |  |
| A | 12 | 44 |
| D | 15 | 56 |
| Abbreviations: M= male, F= female, LC/A= large cell/anaplastic, A= alive, D= dead. ***** Risk group in children >3 years of age. | | |
