# Supplementary material for: Overcoming multiple drug resistance mechanisms in medulloblastoma
Source: Acta Neuropathol Commun. 2014 May 30;2:57. doi: 10.1186/2051-5960-2-57 (PMC4229867; doi:10.1186/2051-5960-2-57)
Supplement: Supplementary file 2 — Additional file 2: Table S2: Antibodies used in the IHC analysis. (DOCX 15 KB) [file 40478_2014_133_MOESM2_ESM.docx]

**Additional file 2: Table S2** Antibodies used in the IHC analysis

| **Primary Ab** | **Supplier** | **Concentration** | **Antigen retrieval** | **Incubation conditions** | **Staining pattern** | **Positive control** |
| --- | --- | --- | --- | --- | --- | --- |
| ABCB1  (anti-C219) | Calbiochem | 1:40 | Pressure cooker | 4 °C overnight | Membranous | Liver |
| β-catenin | Cell signalling | 1:500 | Pressure cooker | 1 hour room temperature | Nuclear and cytoplasmic | Colon |
| GLI1 | Abcam | 1:50 | Steamer | 1 hour room temperature | Cytoplasmic and membranous | Colon |
| NPR3 | Abcam | 1:200 | Steamer | 1 hour room temperature | Membranous | Foetal Brain |
| KCNA1 | Abcam | 1:500 | Steamer | 1 hour room temperature | Membranous | U87 orthotopic xenograft tumour |
| MGMT | Millipore | 1:50 | Steamer | 1 hour room temperature | Nuclear | Tonsil, MB |
| Ki67 | Dako Cytomation | 1:50 | Steamer | 1 hour room temperature | Nuclear | Tonsil |
