# Supplementary material for: Overcoming multiple drug resistance mechanisms in medulloblastoma
Source: Acta Neuropathol Commun. 2014 May 30;2:57. doi: 10.1186/2051-5960-2-57 (PMC4229867; doi:10.1186/2051-5960-2-57)
Supplement: Supplementary file 3 — Additional file 3: Table S3: Correlation of ABCB1 expression with clinicopathological characteristics of DKFZ + Nottingham TMA cohorts. (DOCX 15 KB) [file 40478_2014_133_MOESM3_ESM.docx]

| **Additional file 3: Table S3** Correlation of ABCB1 expression with clinicopathological characteristics of DKFZ + Nottingham TMA cohorts | | | | | | |
| --- | --- | --- | --- | --- | --- | --- |
|  | **ABCB1 immunonegative** | | **ABCB1 immunopositive** | | **Complete cohort** | **Fisher’s exact test** |
| **Variable** | **No.** | **%** | **No** | **%** | **No. (%)** | ***P* value** |
| **Age, years** |  |  |  |  |  |  |
| ≤ 3 | 26 | 18 | 22 | 20 | 49 (19) | *P*= 0.7 |
| >3 | 122 | 81 | 89 | 80 | 210 (81) |  |
| Unknown | 0 | 0 | 1 | 1 | 1 (0.3 ) |  |
| **Gender** |  |  |  |  |  |  |
| Male | 99 | 67 | 66 | 58 | 166 (64) | *P*= 0.24 |
| Female | 49 | 33 | 45 | 42 | 94 (36) |  |
| **Recurrence** |  |  |  |  |  |  |
| No | 94 | 65 | 78 | 70 | 173 (67) | *P*= 0.37 |
| Yes | 51 | 35 | 33 | 30 | 84 (33) |  |
| **Resection status** |  |  |  |  |  |  |
| Total | 94 | 64 | 66 | 60 | 161 (62) | *P*= 0.43 |
| Subtotal | 52 | 35 | 45 | 40 | 97 (37) |  |
| Unknown | 2 | 1 | 0 | 0 | 2 (1) |  |
| **Risk group*** |  |  |  |  |  |  |
| Standard | 50 | 38 | 24 | 27 | 75 (36) | **P= 0.04** |
| High | 71 | 62 | 65 | 73 | 135 (64) |  |
| *Risk group includes children > 3 years of age . | | | | | | |
