# Supplementary material for: Overcoming multiple drug resistance mechanisms in medulloblastoma
Source: Acta Neuropathol Commun. 2014 May 30;2:57. doi: 10.1186/2051-5960-2-57 (PMC4229867; doi:10.1186/2051-5960-2-57)
Supplement: Supplementary file 4 — Additional file 4: Table S4: Clinical characteristics of the 5 patients from whom 6 new MB cell lines were derive. (DOC 39 KB) [file 40478_2014_133_MOESM4_ESM.doc]

**Additional file 4: Table S4** Clinical characteristics of the 5 patients from whom 6 new MB cell lines were der

| **Cell line** | **Age (months)** | **Diagnosis** | **Sex** | **Surgical resection status** | **Treatment** | **Follow up (months)** | **Status** | **Metastatic status** | **MB subtypes** | **MGMT** | **ABCB1 expression**% |
| --- | --- | --- | --- | --- | --- | --- | --- | --- | --- | --- | --- |
| MED 1 | 41 | LC/A MB | M | Partial | CT/RT | 8 | D | M0 (M2 at relapse) | Group 4 | - | 11.2 |
| MED 3 | 118 | Classical | F | complete | CT/ RT | 42 | A | M0 | Group 3 | + | 0 |
| MED4 | 90 | LC/A MB | F | partial | CT | 4 | D | M0 | Group 3 | + | 1.9 |
| MED4R | 94 | Recurrent  LC/A MB | F | Partial | CT | 20 | D | M0 | Group 3 | + | 4.5 |
| MED5R | 57 | Recurrent  LC/A MB | M | partial | CT/RT | 21 | D | M2 | WNT* | + | 6.2 |
| MED6 | 105 | Classical | M | complete | CT/RT | 16 | A | M0 | WNT* | + | 1.2 |
| Abbreviation: LC/A= large cells/anaplastic, M = male, F= female, CT = chemotherapy, RT = radiotherapy, D = dead, A = alive, WNT = β-catenin positive, Group 3= NPR3 positive, Group 4 = KCNA1 positive, MED4 and MED4R are from the same patient. IHC was used to determine the molecular subtype, percentage of ABCB1 expressing cells and MGMT status in each primary tumour. *β-catenin is mutated. | | | | | | | | | | | |
