# Supplementary figures and images for: Overcoming multiple drug resistance mechanisms in medulloblastoma
Source: Acta Neuropathol Commun. 2014 May 30;2:57. doi: 10.1186/2051-5960-2-57 (PMC4229867; doi:10.1186/2051-5960-2-57)

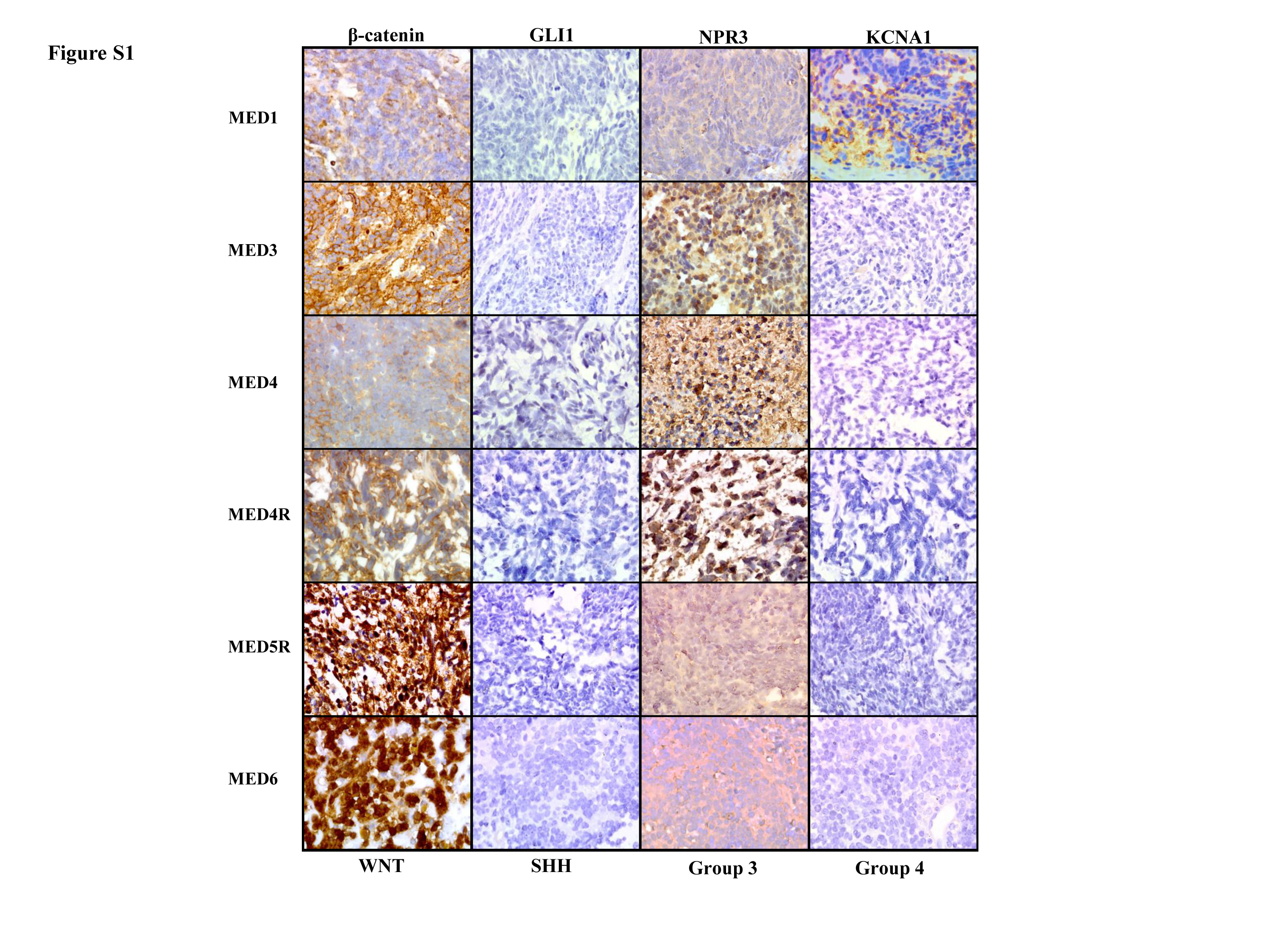

Supplement: Supplementary file 5 — Additional file 5: Figure S1: Patterns of MB marker subtype immunostaining on each patient’s original tissue sample. (JPEG 1 MB) [file 40478_2014_133_MOESM5_ESM.jpeg]

Figure S2

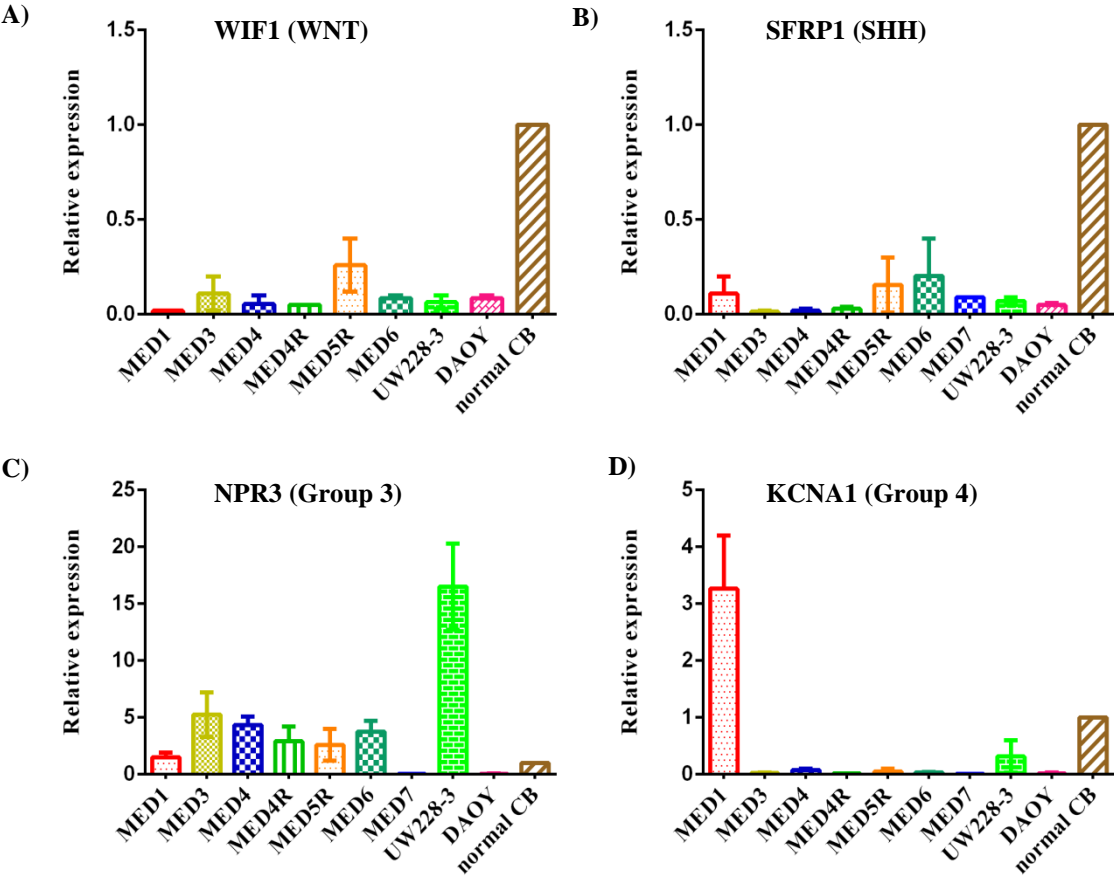

Supplement: Supplementary file 6 — Additional file 6: Figure S2: Molecular sub-classification of the 8 MB cell lines. (PDF 160 KB) [file 40478_2014_133_MOESM6_ESM.pdf]

Figure S3

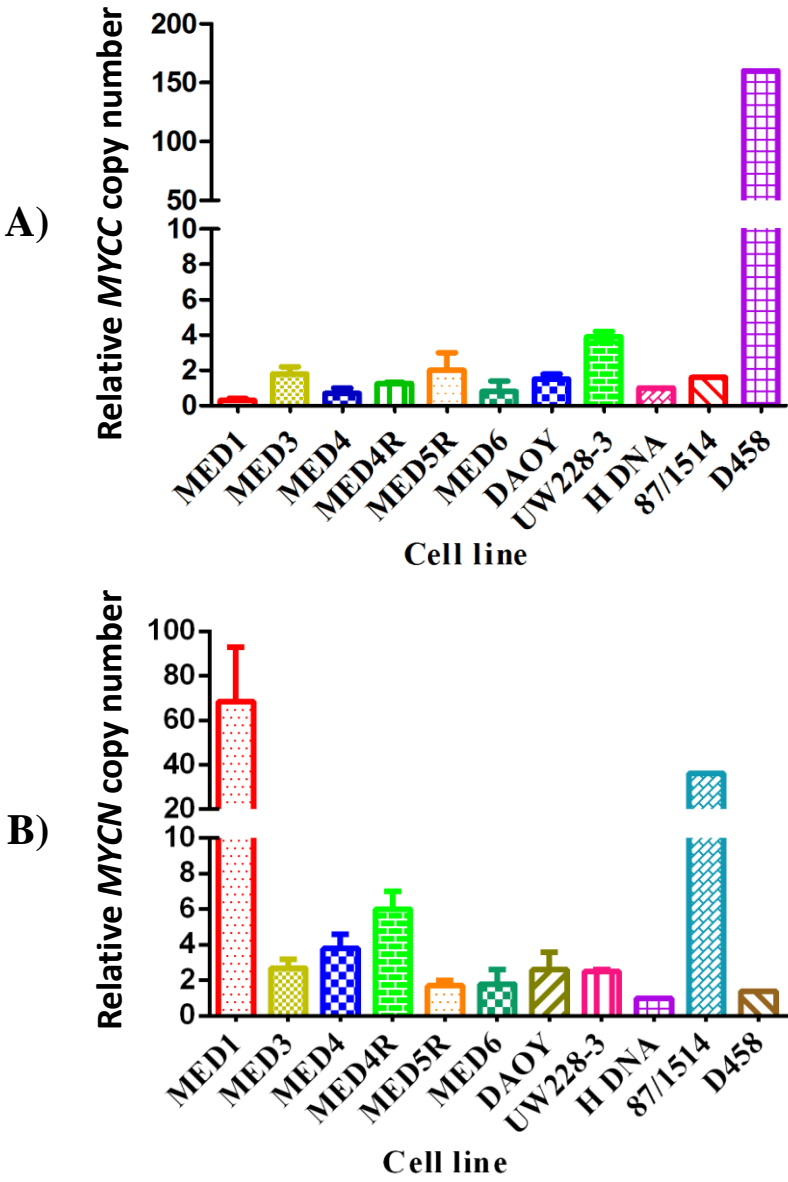

Supplement: Supplementary file 7 — Additional file 7: Figure S3: Elevated MYCC and MYCN copy numbers in MB cell lines. (PDF 323 KB) [file 40478_2014_133_MOESM7_ESM.pdf]

Figure S4

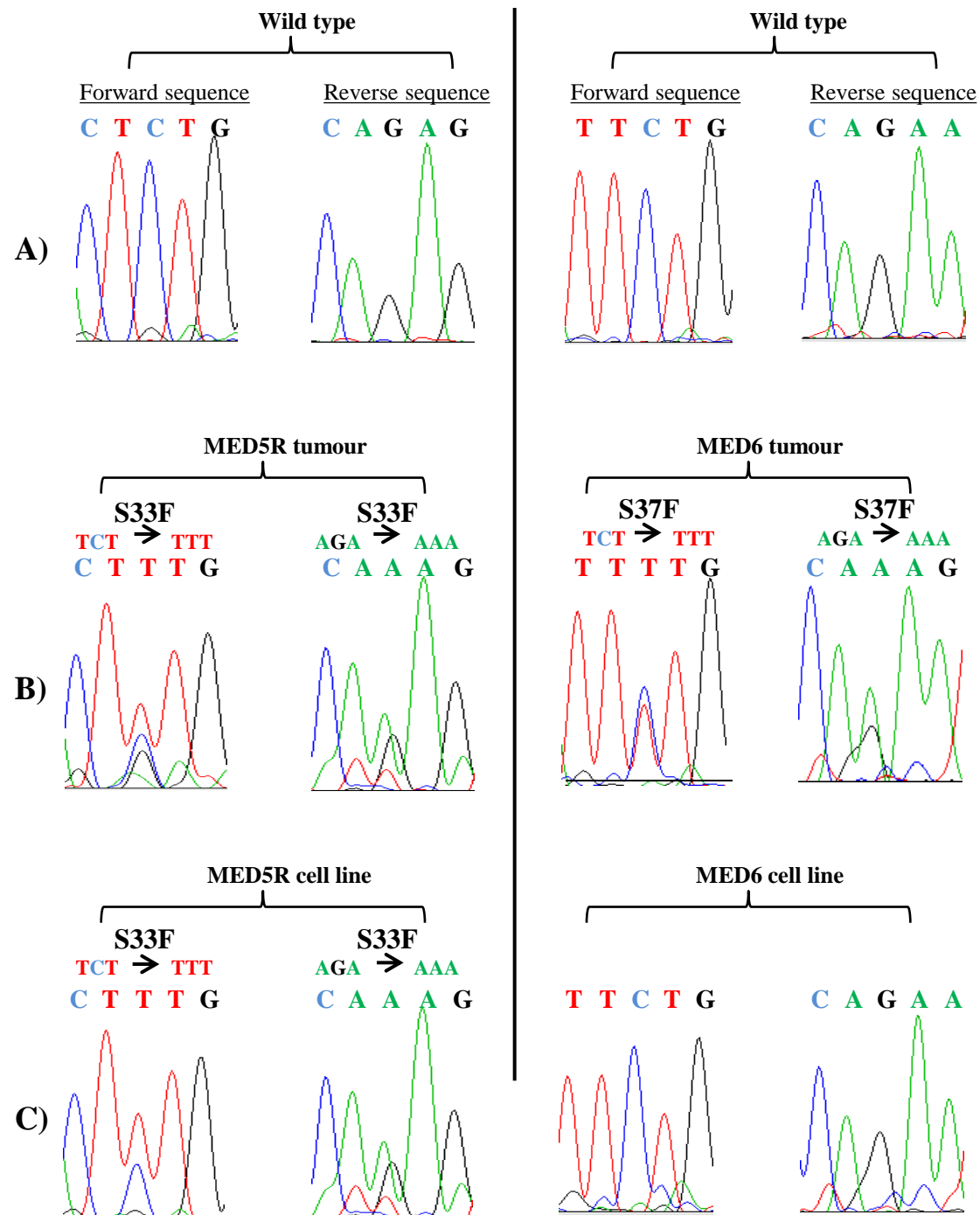

Supplement: Supplementary file 8 — Additional file 8: Figure S4: β-catenin (CTNNB1) sequencing in MED5R and MED6 tumours and cell lines. (PDF 32 KB) [file 40478_2014_133_MOESM8_ESM.pdf]

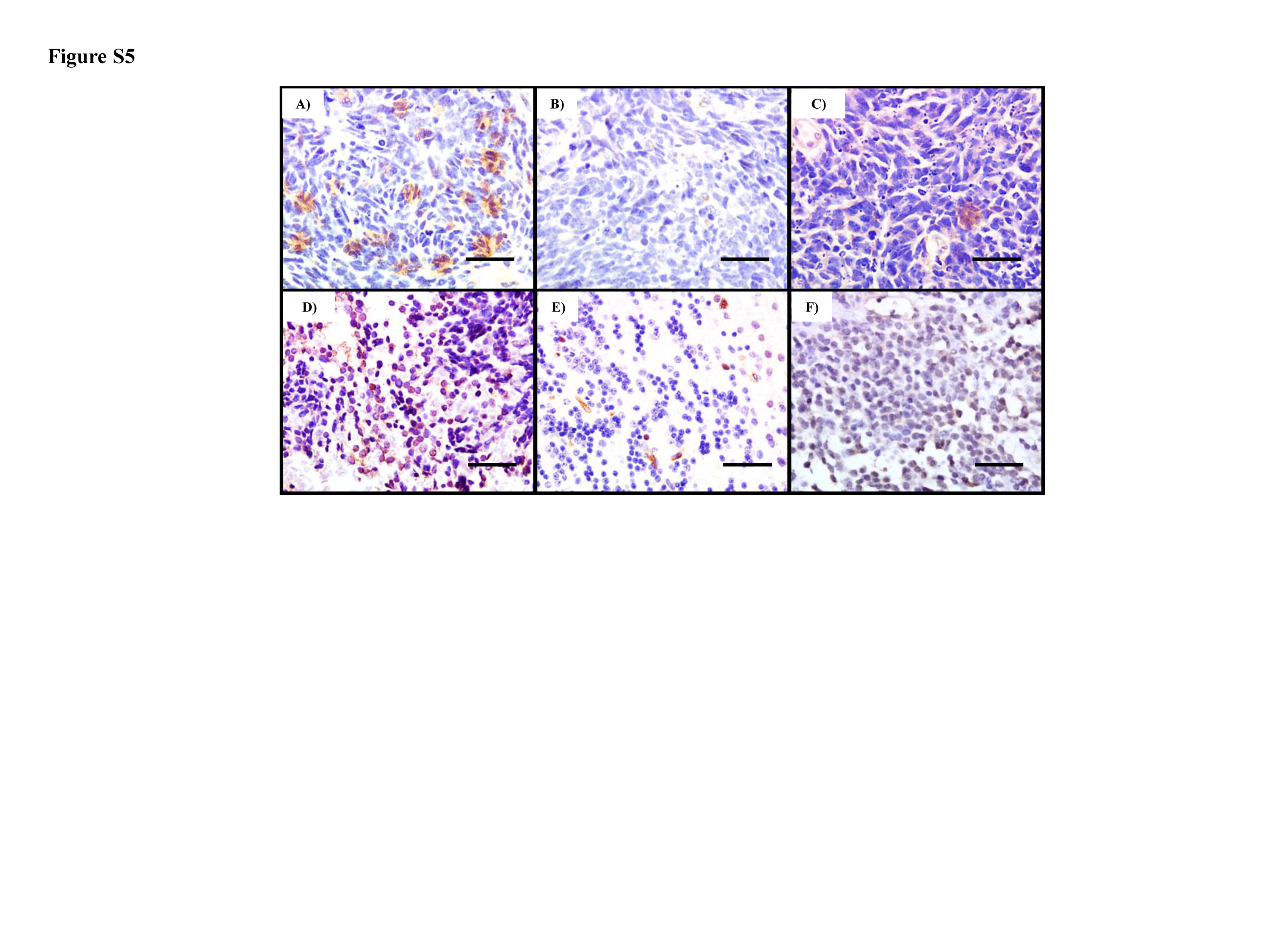

Supplement: Supplementary file 9 — Additional file 9: Figure S5: ABCB1 expression in original patient tumours. (JPEG 695 KB) [file 40478_2014_133_MOESM9_ESM.jpeg]

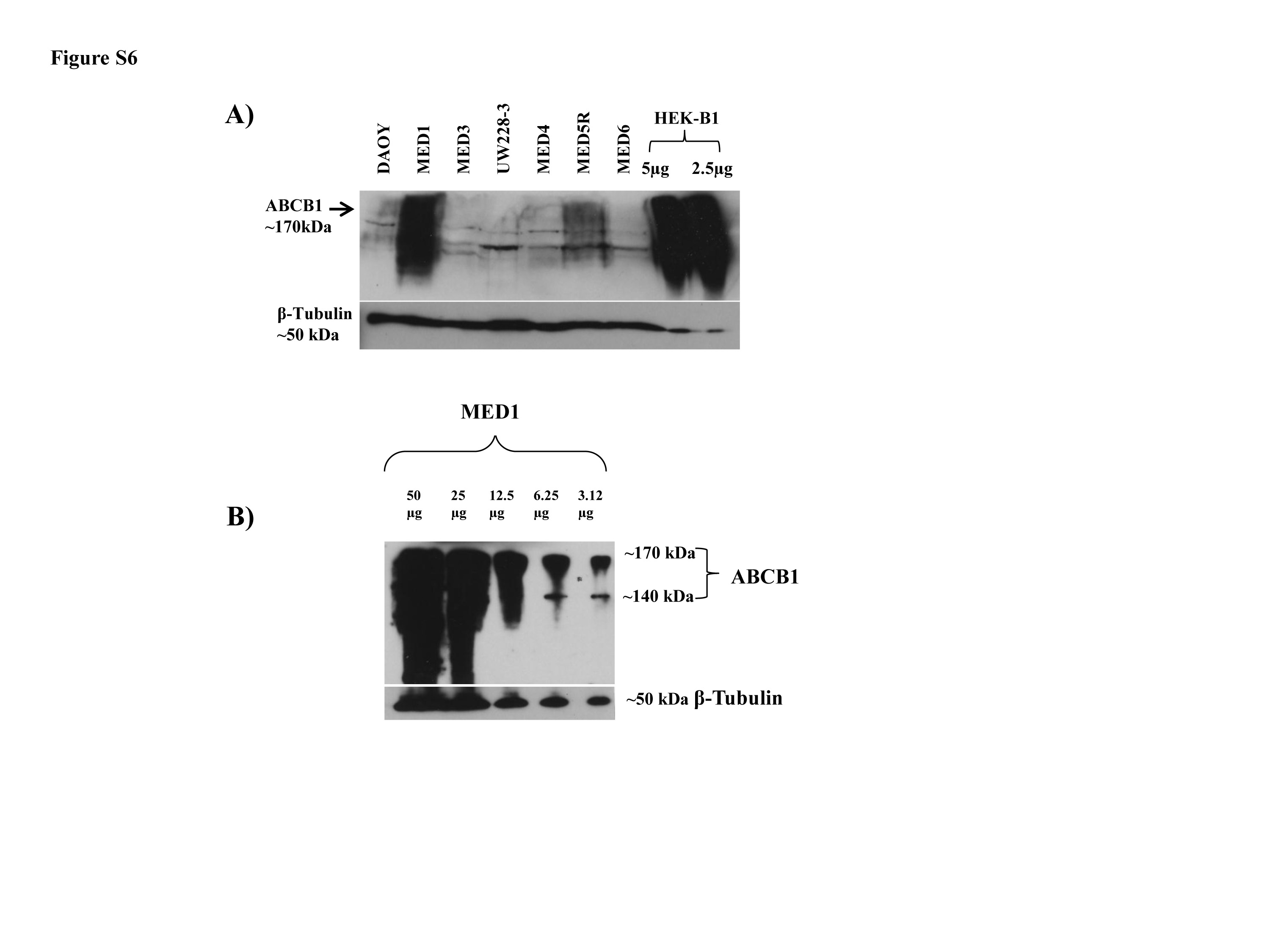

Supplement: Supplementary file 10 — Additional file 10: Figure S6: MED1 cells show high ABCB1 protein expression. (JPEG 185 KB) [file 40478_2014_133_MOESM10_ESM.jpeg]

**Figure S7**

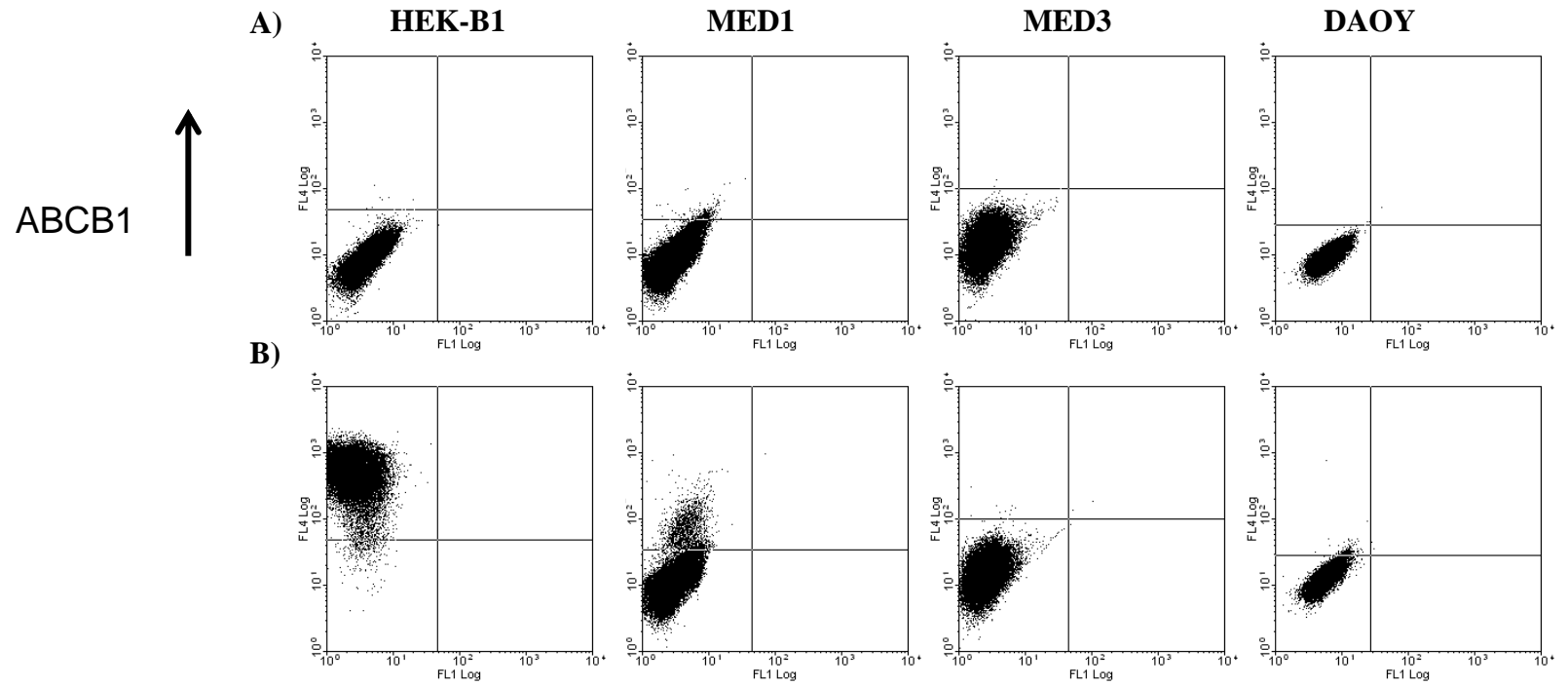

Supplement: Supplementary file 11 — Additional file 11: Figure S7: ABCB1 expression is found in a small subpopulation of cells. (PDF 37 KB) [file 40478_2014_133_MOESM11_ESM.pdf]

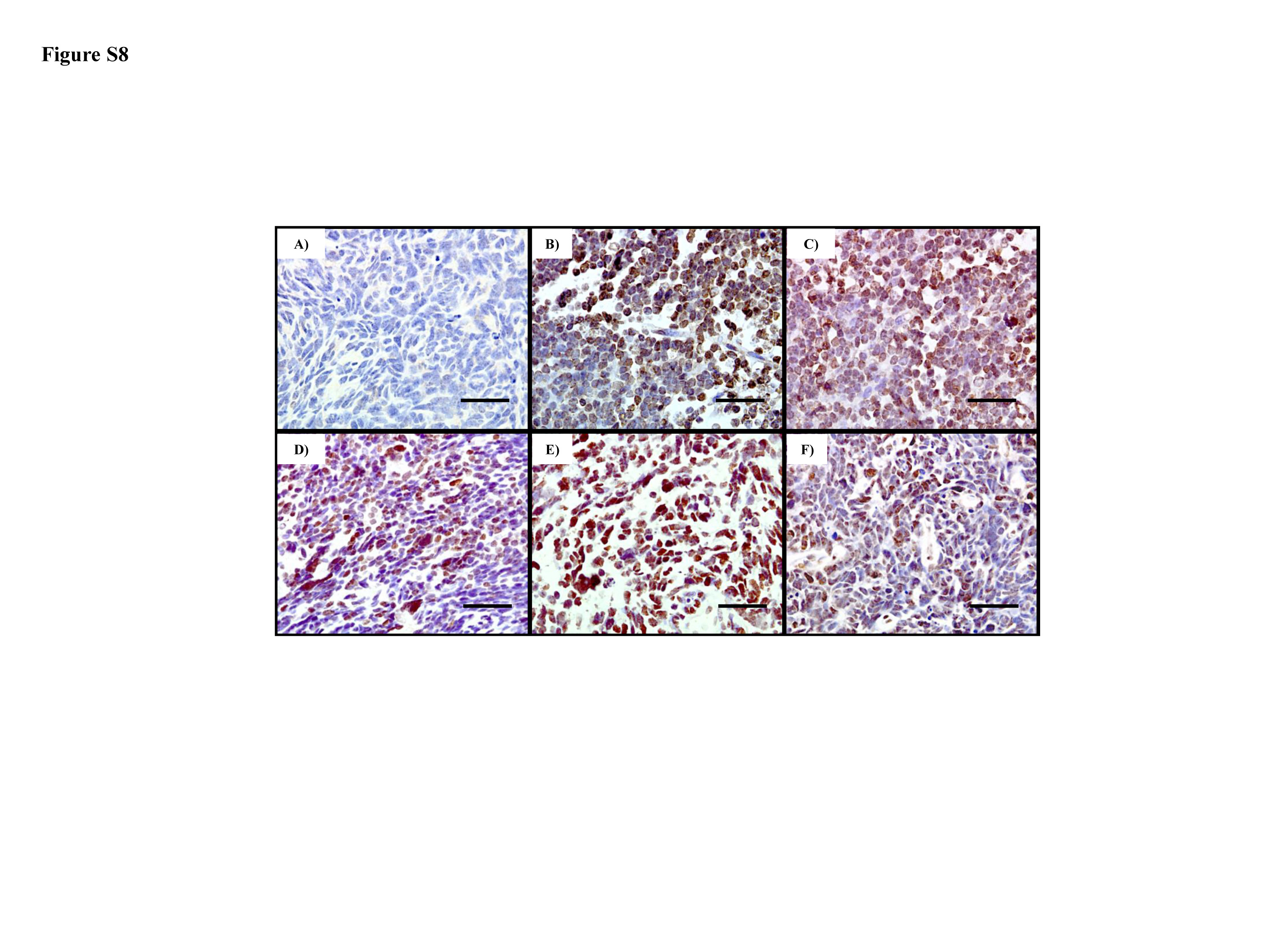

Supplement: Supplementary file 12 — Additional file 12: Figure S8: MGMT expression in the original patient tumours. (JPEG 768 KB) [file 40478_2014_133_MOESM12_ESM.jpeg]

Figure S9

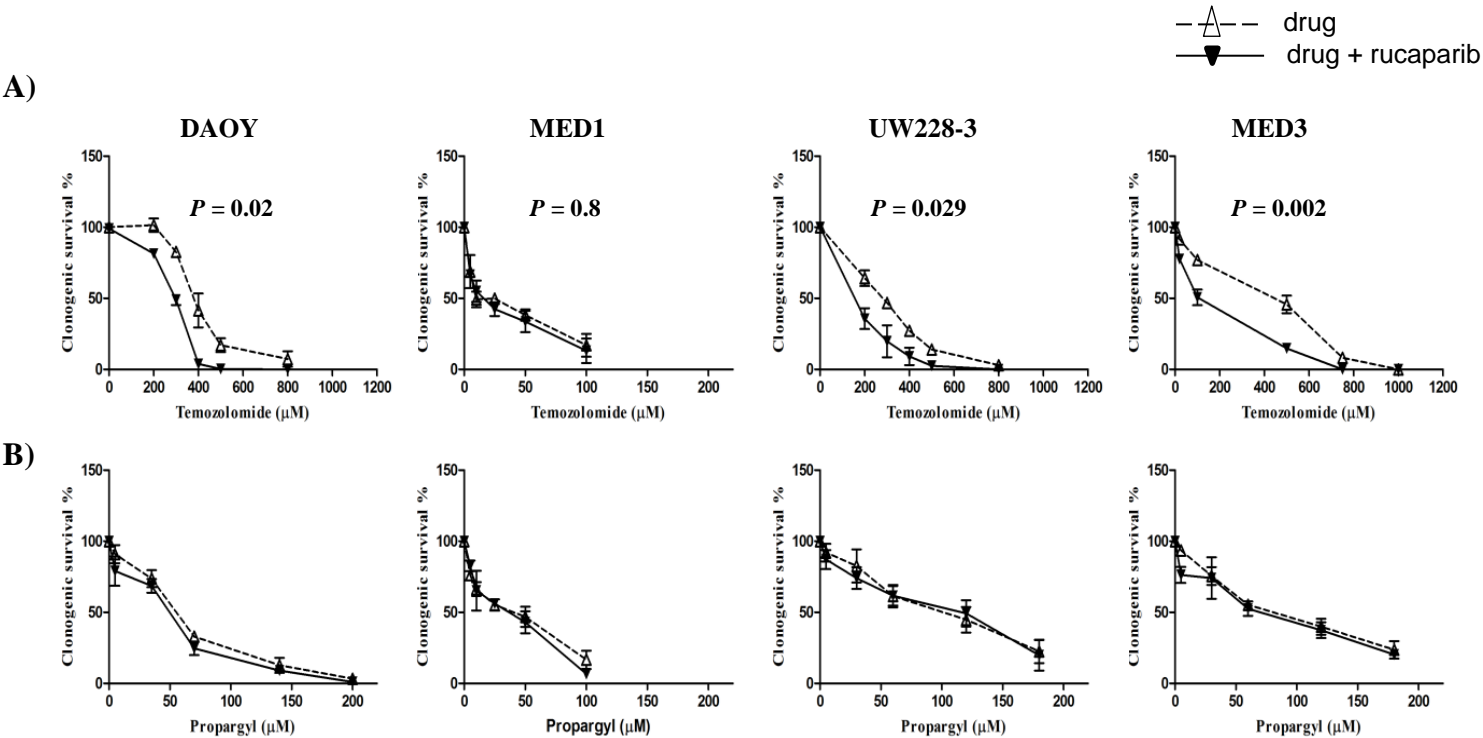

Supplement: Supplementary file 13 — Additional file 13: Figure S9: Chemosensitization of TMZ and propargyl by rucaparib in MB cell lines. (PDF 133 KB) [file 40478_2014_133_MOESM13_ESM.pdf]
